# Targeting Glioblastoma Stem Cells: Therapeutic Strategies and Clinical Perspectives

**DOI:** 10.3390/cancers18091353

**Published:** 2026-04-24

**Authors:** Harikrishna Reddy Rachamala, Sonia Devi Lourembam, Debabrata Mukhopadhyay, Ramcharan Singh Angom

**Affiliations:** 1 Department of Biochemistry and Molecular Biology, Mayo Clinic College of Medicine, Jacksonville, FL 32224, USA; rachamala.hari@mayo.edu; 2 Department of Transplant Immunology and Immunogenetics, All India Institute of Medical Science, Delhi 110029, India

**Keywords:** glioblastoma, glioblastoma stem cells, targeted therapy, tumor microenvironment, precision oncology, cancer stem cell niche, therapy resistance

## Abstract

With limited therapeutic progress despite aggressive multimodal treatment, glioblastoma (GBM) remains one of the deadliest primary brain tumors. Emerging evidence suggests that GSCs are key drivers of tumor initiation, intratumoral heterogeneity, therapeutic resistance, and recurrence. GSCs retain self-renewal capacity, multilineage differentiation potential, and remarkable plasticity, enabling them to adapt to diverse microenvironmental cues. These properties are upheld by dysregulated developmental and oncogenic signaling pathways, including Notch, Wnt/β-catenin, Hedgehog, PI3K/AKT/mTOR, and STAT3, as well as epigenetic and metabolic reprogramming. In addition, dedicated niches such as hypoxic and perivascular microenvironments critically support GSC maintenance and immune evasion. In this review, we summarize the current understanding of the molecular pathways governing GSC biology, examine their interactions with the tumor microenvironment, and discuss emerging therapeutic strategies targeting GSCs, including pathway inhibition, differentiation therapy, immunotherapy, and nanomedicine-based drug delivery. We highlight key challenges and future directions for translating GSC-targeted approaches into effective clinical interventions for GBM.

## Simple Summary

Glioblastoma (GBM) remains a highly aggressive brain tumor with poor clinical outcomes despite intensive treatment approaches. Glioblastoma stem cells (GSCs) play a pivotal role in tumor initiation, heterogeneity, therapy resistance, and recurrence due to their self-renewal, differentiation capacity, and plasticity. These properties are driven by dysregulated signaling pathways and supported by specialized tumor microenvironments. This review stresses key molecular mechanisms underlying GSC biology, their interactions with the tumor niche, and emerging therapeutic strategies targeting GSCs to improve GBM treatment outcomes.

## 1. Introduction

Glioblastoma (GBM) represents the most aggressive and common primary malignant tumor of the adult central nervous system, owing to its rapid progression and extensive heterogeneity [1,2,3]. Despite standard therapy, glioblastoma patients have a median survival of ~14–18 months and a 5-year survival below 10% [1,2]. The breakthrough trial led by Roger Stupp instituted the present standard of care, yet therapeutic improvements over the past two decades have been modest. Moreover, tumor recurrence, within months of initial therapy, highlights the pressing need for more efficient treatment strategies.

Toward this, molecular profiling efforts, including those from The Cancer Genome Atlas (TCGA), have discovered extensive inter- and intratumoral heterogeneity in GBM [4,5,6], characterized by varied genetic alterations such as EGFR amplification and mutation, PTEN loss, TP53 mutation, and aberrant activation of receptor tyrosine kinase (RTK)/RAS/PI3K pathways [4,7]. These studies highlight the molecular complexity of GBM. However, targeted therapies directed against these alterations have yielded limited clinical benefit, suggesting that additional cellular and microenvironmental mechanisms sustain tumor growth and therapeutic resistance.

**Figure 1:**
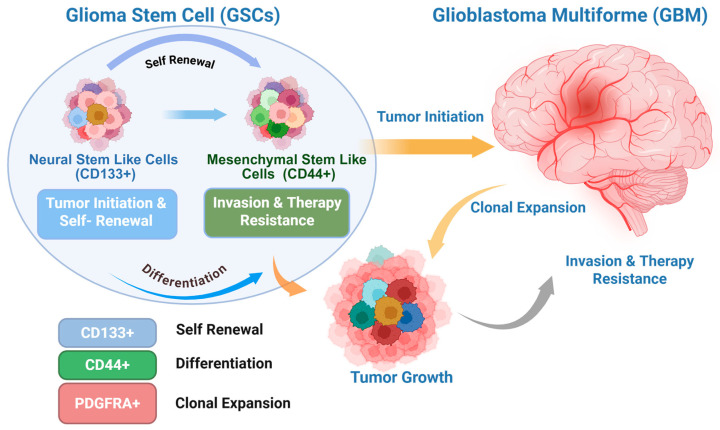
Glioma stem cell (GSC) heterogeneity drives glioblastoma progression. Schematic representation illustrates the role and diversity of GSCs in glioblastoma multiforme (GBM). GSCs comprise distinct subpopulations, including neural stem-like (CD133^+^), mesenchymal-like (CD44^+^), and oligodendrocyte precursor-like (PDGFRA^+^) cells. These populations contribute differentially to tumor biology: CD133^+^ cells drive tumor initiation and self-renewal, CD44^+^ cells promote invasion and therapy resistance, and PDGFRA^+^ cells support proliferation and tumor growth. The dynamic interplay among these subpopulations underlies tumor heterogeneity and progression in glioblastoma (GBM). Neural stem-like GSCs (CD133^+^; shown in blue) are primarily associated with self-renewal and tumor initiation, whereas mesenchymal stem-like GSCs (CD44^+^; shown in green) contribute to invasion and therapy resistance. The red/pink tumor mass represents clonal expansion and tumor growth driven by heterogeneous cell populations. Multicolored cell clusters highlight intratumoral heterogeneity. Orange arrows indicate key processes of tumor progression, including initiation, clonal expansion, and invasion, while grey/black arrows depict dynamic transitions between GSC states, such as self-renewal and differentiation. PDGFRA^+^ populations (indicated in red labeling) reflect proliferative/proneural lineage contributions to tumor expansion. The image was created in BioRender.com.

Emerging evidence identified a subpopulation of tumor cells with stem-like properties that drive tumor initiation, progression, and recurrence [8,9,10] (Figure 1). These glioblastoma stem cells (GSCs) are defined functionally by their ability to self-renew, differentiate into multiple neural lineages, and recapitulate the original tumor upon xenotransplantation. Early studies identified CD133 (Prominin-1) as a marker for GSCs, although subsequent work demonstrated that GSCs may also exist within CD133-negative populations, reflecting substantial phenotypic plasticity [11,12]. GSC plasticity enables dynamic state transitions that drive therapeutic resistance, including enhanced DNA damage responses, efficient repair, drug efflux, and apoptosis resistance [8,13]. Additionally, GSCs often reside in specialized microenvironmental niches, including hypoxic and perivascular regions that provide critical signals for stemness maintenance and immune evasion. Hypoxia-inducible factors (HIFs) promote the expression of stemness-associated genes, while endothelial-derived signals within the perivascular niche further reinforce self-renewal programs [14,15]. These niche interactions establish a supportive ecosystem that protects GSCs from cytotoxic therapies. At the molecular level, GSC maintenance is sustained by aberrant activation of developmental and oncogenic signaling pathways, including Notch, Wnt/β-catenin, Hedgehog, PI3K/AKT/mTOR, and STAT3 cascades. These pathways coordinate transcriptional networks governing self-renewal, proliferation, metabolic adaptation, and survival. Furthermore, epigenetic regulation also contributes to GSC identity, with chromatin remodeling complexes, DNA methylation patterns, and non-coding RNAs determining stemness-associated gene expression programs [16,17,18]. Moreover, metabolic reprogramming enables GSCs to flexibly switch between glycolysis and oxidative phosphorylation, enhancing their capacity to survive under fluctuating oxygen and nutrient conditions [19]. The tumor microenvironment (TME) also plays a central role in sustaining GSC populations. Interactions with tumor-associated macrophages, microglia, endothelial cells, and extracellular matrix components create an immunosuppressive and pro-stemness milieu [20]. Immune checkpoint expressions, including PD-L1, and secretion of immunomodulatory cytokines contribute to immune evasion. Emerging data suggest that bidirectional communication between GSCs and stromal components not only maintains stemness but also drives tumor evolution and heterogeneity [21]. Given GSCs’ underlying role in tumor maintenance and recurrence, targeting these cells has surfaced as a promising therapeutic strategy. Currently, approaches under examination comprise inhibition of stemness-associated signaling pathways, differentiation therapy, metabolic targeting, immunotherapeutic strategies such as CAR-T cells and oncolytic viruses, and nanomedicine-based drug delivery systems designed to overcome the blood–brain barrier. However, challenges such as cellular plasticity, pathway redundancy, toxicity to normal neural stem cells, and limited drug penetration into the brain persist as barriers to clinical translation [22]. In this review, we summarize the current understanding of the molecular and cellular mechanisms governing GSC biology, examine the role of specialized microenvironmental niches in sustaining stemness and therapeutic resistance, and discuss emerging therapeutic strategies to eradicate GSCs. We also highlight critical challenges and future directions needed to translate GSC-targeted approaches into durable, effective treatments for GBM.

## 2. Biological Characteristics of Glioblastoma Stem Cells

### 2.1. Definition and Identification of GSCs

The concept of GSCs arises from the broader cancer stem cell (CSC) hypothesis, which proposes that a subset of tumor cells possesses stem-like properties, including self-renewal, multilineage differentiation, and tumor-propagating capacity. In glioblastoma, seminal work by Singh and colleagues demonstrated that CD133-positive tumor cells isolated from patient specimens could initiate tumors in immunodeficient mice that recapitulated the original histopathology, whereas CD133-negative cells exhibited markedly reduced tumorigenicity [9]. These findings provided functional evidence for a stem-like tumor-propagating population in GBM.

#### 2.1.1. Stemness Markers

CD133 (Prominin-1) developed as one of the earliest markers enriched in GSCs. CD133^+^ cells exhibit higher neurosphere-forming capacity, boosted expression of stemness-associated genes, and increased tumor-initiating capacity in xenograft models [8,9]. However, subsequent studies demonstrated that CD133^−^ populations can also display tumor-propagating capacity, underscoring the phenotypic heterogeneity of GSCs and indicating that CD133 is not an exclusive marker for GSCs [11,12]. These findings established that CD133 should be considered an enrichment marker rather than a definitive identifier of stemness.

Beyond CD133, a broader repertoire of transcriptional and surface markers has been implicated in defining GSC populations (Table 1). SOX2, a master regulator of pluripotency and neural stem cell identity, is consistently expressed across GSC subsets and plays a central role in maintaining self-renewal and tumorigenic capacity. Functional studies have shown that SOX2 depletion impairs sphere formation, reduces proliferation, and diminishes tumor propagation in vivo, highlighting its essential role in sustaining stem-like states [16,23]. Mechanistically, SOX2 integrates signals from developmental pathways such as Notch and Wnt to regulate transcriptional programs associated with stemness and lineage commitment. Nestin, an intermediate filament protein characteristic of neural progenitor cells, is widely expressed in GBM and is particularly enriched in stem-like compartments. Its expression reflects an undifferentiated, proliferative state and is frequently used in combination with other markers to identify GSC populations. Similarly, OLIG2, a transcription factor essential for oligodendrocyte lineage specification, marks a proliferative progenitor-like population in GBM and contributes to tumor growth and lineage plasticity [24]. OLIG2 has also been shown to regulate cell cycle progression and suppress p53-mediated differentiation, thereby reinforcing tumorigenic potential. Additional markers such as CD44, integrin α6 (ITGA6), L1CAM, and A2B5 further highlight the diversity of GSC phenotypes. CD44 is commonly associated with mesenchymal-like GSCs and is linked to invasive behavior, therapeutic resistance, and interaction with the extracellular matrix [25]. Integrin α6 mediates adhesion to the vascular niche and supports GSC maintenance through interactions with laminin-rich microenvironments [26,27]. L1CAM has been shown to promote GSC survival and radioresistance by activating DNA damage response pathways, while A2B5 marks a progenitor-like population with tumor-initiating capacity [28,29].

Importantly, single-cell transcriptomic analyses have revealed that GSCs comprise cell signatures including proneural, mesenchymal, and astrocyte-like programs [17]. These states are highly dynamic and influenced by microenvironmental cues such as hypoxia, inflammation, and therapeutic stress. For example, hypoxic conditions promote a shift toward a stem-like phenotype by activating hypoxia-inducible factors (HIFs). At the same time, exposure to radiation or chemotherapy can induce dedifferentiation of non-stem tumor cells into stem-like states [8,15,16]. Collectively, these findings indicate that no single marker is sufficient to define GSCs. Instead, GSC identity is best understood as a functional and dynamic cellular state governed by transcriptional, epigenetic, and microenvironmental regulation. This conceptual shift from static marker-based identification to state-based characterization has important implications for therapeutic targeting, underscoring the need to disrupt regulatory networks and niche interactions that sustain stemness rather than relying solely on marker-directed strategies.

#### 2.1.2. Functional Validation of Glioblastoma Stem Cells: Assays for Self-Renewal, Tumor Initiation, and Cellular Plasticity

Surface markers alone are insufficient to accurately define GSCs, and functional assays remain the primary method for establishing stem-like properties. These assays directly assess the defining features of GSCs—self-renewal, multipotency, and tumor-initiating capacity—providing a rigorous framework for identifying tumor-propagating populations [9,26]. These assays include the neurosphere formation assay, which evaluates the ability of single cells to generate primary spheres and, critically, to form secondary and tertiary spheres upon serial passaging, which is indicative of self-renewal capacity [9,33]. However, one limitation here is that a progenitor cell may also form a sphere with restricted proliferative potential, and cell aggregation can artificially inflate sphere counts if clonal conditions are not strictly maintained [34]. Therefore, overcoming these limitations, using in vivo tumor initiation assays remains the most rigorous functional test of GSC identity. Regulating dilution transplantation into immunocompromised mice, such as NOD/SCID or NSG strains, permits quantification of tumor-propagating frequency [8,9]. Prominently, orthotopic xenograft models, in which tumor cells are implanted into the brain, provide a more physiologically relevant environment compared to subcutaneous models by preserving interactions with neural, vascular, and immune components of the tumor microenvironment [35,36]. Patient-derived xenograft (PDX) models have further advanced the study of GSC biology by maintaining genomic integrity and intertumoral heterogeneity across serial passages [37]. In parallel, three-dimensional glioblastoma organoid systems have emerged as powerful ex vivo platforms that recapitulate the spatial architecture, hypoxic gradients, and lineage hierarchies observed in patient tumors [38,39]. Enabling the investigation of GSC dynamics and therapeutic responses in a controlled yet physiologically relevant context.

More recently, lineage tracing and genetic barcoding approaches have allowed tracking of individual cell populations possible over time [17,40]. Integration of single-cell transcriptomics with functional assays has further demonstrated that stem cell represents a dynamic cellular state [17,41]. Despite these advances, each functional assay has inherent limitations. In vitro systems lack the full complexity of the tumor microenvironment, whereas in vivo models are constrained by species-specific differences and the absence of a fully competent immune system. Therefore, a combinatorial approach integrating multiple functional assays with molecular profiling is essential for precisely defining GSC populations. Collectively, GSC identity is best defined by functional behavior rather than static marker expression.

### 2.2. Mechanisms of Therapy Resistance

One of the defining characteristics of GSCs is their pronounced resistance to standard therapies, including temozolomide chemotherapy and radiotherapy.

#### 2.2.1. Enhanced DNA Damage Response and Genome Protection in Glioblastoma Stem Cells

GSCs exhibit a highly augmented DNA damage response (DDR), which plays a central role in their resistance to genotoxic therapies such as ionizing radiation and alkylating chemotherapy. Following irradiation, CD133^+^ GSCs preferentially activate checkpoint kinases, including Chk1 and Chk2, facilitating rapid cell cycle arrest and efficient repair of DNA double-strand breaks (DSBs) [8]. This improved checkpoint activation permits GSCs to maintain genomic integrity while avoiding mitotic catastrophe. Robust activation of the ATM (ataxia telangiectasia mutated) and ATR (ATM and Rad3-related) signaling pathways coordinate DNA damage sensing, checkpoint enforcement, and repair. GSCs exhibit elevated basal and inducible levels of DDR components, including γH2AX, RAD51, BRCA1/2, and DNA-dependent protein kinase (DNA-PK), promoting efficient repair through both homologous recombination (HR) and non-homologous end joining (NHEJ) pathways [13,42]. Preferential utilization of HR, a high-fidelity repair mechanism, contributes to the accurate resolution of DSBs and enhances cell survival following radiation-induced damage. In addition to canonical DDR signaling, GSCs exhibit increased activity of replication stress response pathways, including ATR-Chk1 signaling, which stabilizes stalled replication forks and prevents fork collapse under therapeutic stress [43,44]. This is particularly relevant in rapidly proliferating tumor cells, where replication-associated DNA damage is a major source of genomic instability. By efficiently managing replication stress, GSCs maintain proliferative capacity while avoiding lethal DNA lesions. Epigenetic regulation further reinforces DDR efficiency in GSCs. Chromatin remodeling factors and histone modifications facilitate rapid access of repair machinery to damage DNA. For example, histone acetylation and methylation states influence the recruitment of repair complexes such as 53BP1 and BRCA1, thereby modulating pathway choice between NHEJ and HR [16]. Moreover, transcriptional programs driven by stemness-associated factors, including SOX2 and OLIG2, have been linked to the regulation of DDR gene expression, suggesting a direct coupling between stem cell identity and genome maintenance mechanisms. Importantly, the enhanced DDR in GSCs is closely linked to their quiescent or slow-cycling state. A subset of GSCs resides in a relatively dormant state, thereby reducing the accumulation of replication-associated DNA damage and increasing resistance to therapies that preferentially target proliferating cells [10]. Upon exposure to stress, these cells can re-enter the cell cycle and repopulate the tumor, contributing to recurrence. Therapeutically, targeting DDR pathways in GSCs has emerged as a promising strategy to overcome treatment resistance. Inhibition of key DDR components such as ATM, ATR, Chk1/2, or PARP has been shown to sensitize GSCs to radiation and chemotherapy in preclinical models [28,43]. However, the redundancy and adaptability of DNA repair pathways, along with potential toxicity to normal neural stem cells, remain significant challenges for clinical translation.

#### 2.2.2. Drug Efflux Transporters and Chemoresistance in Glioblastoma Stem Cells

GSCs exhibit pronounced resistance to chemotherapy, in part due to the elevated expression and activity of ATP-binding cassette (ABC) transporters. Among the most extensively studied transporters in GSCs are ABCG2 (also known as breast cancer resistance protein, BCRP) and ABCB1 (P-glycoprotein, MDR1), both of which contribute to multidrug resistance by effluxing chemotherapeutic agents across the plasma membrane [45,46]. At the molecular level, ABC transporter expression in GSCs is tightly regulated by stemness-associated and stress-responsive signaling pathways. Hypoxia, a defining feature of the glioblastoma microenvironment, induces ABCG2 expression by activating hypoxia-inducible factors (HIF-1α and HIF-2α), thereby linking metabolic stress to drug resistance [32]. Additionally, developmental pathways such as Notch, Wnt/β-catenin, and PI3K/AKT signaling have been implicated in the transcriptional regulation of ABC transporters, reinforcing their expression in stem-like states [47]. Clinically, the overexpression of ABC transporters poses a major barrier to effective therapy. In addition to limiting the intracellular accumulation of agents such as temozolomide, these transporters also contribute to the restricted penetration of drugs across the blood–brain barrier (BBB), where ABCB1 and ABCG2 are highly expressed in endothelial cells. This dual role, within both tumor cells and the BBB, creates a formidable pharmacological obstacle. Although several ABC transporter inhibitors have been developed, their clinical success has been limited by toxicity, lack of specificity, and compensatory upregulation of alternative efflux mechanisms [48]. Emerging therapeutic strategies aim to bypass ABC-mediated resistance by using complementary methods, such as nanoparticle-based drug delivery systems, BBB-penetrating carriers, and producing products that evade ABC-mediated efflux. Additionally, targeting upstream regulatory pathways that control ABC transporter expression may provide a more effective means of sensitizing GSCs to chemotherapy. By actively reducing intracellular drug concentrations and integrating with stemness-associated signaling networks, these transporters enable GSC survival under therapeutic pressure and contribute to tumor recurrence.

#### 2.2.3. Quiescence and Metabolic Plasticity in Glioblastoma Stem Cells

A functionally distinct subset of GSCs exists in a quiescent or slow-cycling state, which confers a significant survival advantage under therapeutic pressure. These dormant cells serve as a reservoir for tumor regeneration and are a major driver of disease recurrence [10,26]. Both intrinsic signaling networks and extrinsic microenvironmental cues tightly regulate quiescence in GSCs. Hypoxic niches within the tumor microenvironment play a central role in maintaining dormancy, largely by activating hypoxia-inducible factors (HIF-1α and HIF-2α), which promote stemness and suppress differentiation [32]. In parallel, signaling pathways such as Notch, TGF-β, and BMP contribute to maintaining a quiescent state by modulating cell cycle regulators, including p21, p27, and cyclin-dependent kinases (CDKs). These pathways collectively enforce reversible cell cycle arrest while preserving the capacity for rapid re-entry into proliferation in response to environmental or therapeutic cues [17,49].

In parallel with their cell cycle plasticity, GSCs exhibit remarkable metabolic flexibility, enabling them to adapt to fluctuations in nutrient and oxygen availability. Unlike differentiated tumor cells, which are often predominantly glycolytic (the “Warburg effect”), GSCs can dynamically switch between glycolysis and oxidative phosphorylation (OXPHOS) in response to environmental conditions. Some GSC populations rely heavily on mitochondrial respiration for energy production and demonstrate increased mitochondrial mass, membrane potential, and reactive oxygen species (ROS) buffering capacity [19]. These OXPHOS-dependent cells are often more resistant to radiation and chemotherapy due to enhanced bioenergetic efficiency and antioxidant defenses.

Conversely, under hypoxic conditions, GSCs undergo metabolic reprogramming toward glycolysis, driven by HIF-mediated transcriptional activation of glycolytic enzymes and glucose transporters. This shift allows survival in low-oxygen environments and supports the maintenance of stemness-associated gene expression programs [50]. Additionally, GSCs can utilize alternative metabolic pathways, including fatty acid oxidation and glutamine metabolism, to sustain energy production and biosynthesis under stress conditions. This metabolic adaptability is further supported by interactions with the tumor microenvironment, including metabolic coupling with stromal and immune cells. Emerging evidence also links metabolic state to epigenetic regulation in GSCs. Metabolites such as α-ketoglutarate, acetyl-CoA, and NAD^+^ serve as cofactors for chromatin-modifying enzymes, thereby coupling cellular metabolism to transcriptional control of stemness and differentiation programs. This integration of metabolic and epigenetic regulation further enhances GSC adaptability and contributes to their resistance to therapy. Therapeutically, targeting quiescent and metabolically flexible GSC populations present a significant challenge. Strategies aimed at forcing cell cycle entry, inhibiting key metabolic pathways (e.g., OXPHOS inhibitors or glycolysis blockers), or disrupting niche-derived signals are being explored to sensitize these cells to conventional therapies. However, the redundancy and adaptability of these mechanisms necessitate combinatorial approaches to eliminate this resilient cell population effectively. Understanding the regulatory networks governing these processes is essential for developing durable, effective treatments for glioblastoma. The involvement of α-ketoglutarate in glioblastoma metabolism also highlights the importance of isocitrate dehydrogenase 1 (IDH1) in regulating tumor biology. In glioblastoma, IDH1 is predominantly wild-type (IDH1^wt^), a status associated with increased tumor aggressiveness, enhanced metabolic flexibility under redox stress, and maintenance of epigenetic stability. In contrast to IDH-mutant gliomas, which accumulate the oncometabolite 2-hydroxyglutarate and exhibit widespread epigenetic reprogramming, IDH1^wt^ glioblastomas retain functional α-ketoglutarate-dependent dioxygenase activity, thereby supporting adaptive transcriptional and metabolic responses [51].

Recent longitudinal transcriptomic analyses further underscore the heterogeneity and adaptability of IDH1^wt^ glioblastoma. For example, IDH1^wt^ tumors can be stratified based on their transcriptional response to standard-of-care therapies, with important implications for targeted treatment strategies [52]. Moreover, integrative analyses from large consortia studies have demonstrated that glioma progression is driven by both intrinsic genetic evolution and dynamic interactions with the tumor microenvironment [53]. Collectively, these findings highlight the central role of IDH1 status in shaping metabolic regulation, epigenetic landscape, and therapeutic responsiveness in glioblastoma.

#### 2.2.4. Resistance to Temozolomide and Radiation in Glioblastoma Stem Cells

GSCs exhibit profound resistance to standard-of-care therapies, including temozolomide (TMZ) chemotherapy and ionizing radiation, which collectively contribute to tumor recurrence. TMZ, a DNA-alkylating agent and the backbone of GBM chemotherapy, induces cytotoxic lesions primarily at the O6 position of guanine. The DNA repair enzyme O6-methylguanine-DNA methyltransferase (MGMT) directly reverses these lesions, and elevated MGMT expression in GSCs is strongly associated with intrinsic chemoresistance [54,55]. Even in MGMT-promoter-methylated tumors, GSCs can evade TMZ-induced cytotoxicity by upregulating alternative DNA repair pathways, including base excision repair (BER) and mismatch repair (MMR), and by activating anti-apoptotic and pro-survival signaling cascades [8,10].

Temozolomide resistance in GSCs is further reinforced by their quiescent or slow-cycling state, which reduces the accumulation of replication-associated DNA damage and limits the effectiveness of agents targeting proliferating cells. In addition, the overexpression of ATP-binding cassette (ABC) transporters, such as ABCG2 and ABCB1, actively effluxes TMZ and other chemotherapeutics, further decreasing intracellular drug accumulation [46]. These combined mechanisms enable GSCs to persist following chemotherapy and serve as a reservoir for tumor regrowth. Radioresistance in GSCs is similarly multifactorial. Enhanced activation of DNA damage response (DDR) pathways, including ATM/ATR signaling and homologous recombination repair, allows efficient repair of radiation-induced double-strand breaks [8,56]. GSCs also exhibit elevated antioxidant defenses, including increased glutathione levels and ROS-scavenging enzymes, which mitigate oxidative DNA damage induced by ionizing radiation [19]. Concurrently, pro-survival pathways such as PI3K/AKT, STAT3, and Notch signaling are upregulated in GSCs, promoting cell survival, stemness maintenance, and therapeutic evasion [26,47]. Further, the hypoxic microenvironment of GBM worsens therapy resistance. Hypoxia reduces the formation of oxygen-derived free radicals necessary for DNA damage “fixation” during radiation, diminishing cytotoxicity [32]. Hypoxia also activates HIF-dependent transcriptional programs that enhance DNA repair, promote stemness, induce quiescence, and upregulate ABC transporters, collectively creating a highly resistant GSC niche. Additionally, interactions with perivascular and extracellular matrix components provide survival signals and reinforce resistance phenotypes. GSCs evade both TMZ and radiotherapy by integrating enhanced DNA repair, drug efflux, pro-survival signaling, quiescence, metabolic adaptability, and microenvironmental protection. This resilience underlies the high recurrence rates of GBM and underscores the need for combinatorial therapeutic strategies that simultaneously target stemness, DNA repair, metabolic plasticity, and the microenvironment.

## 3. Core Signaling Networks Governing GSC Maintenance

**Figure 2:**
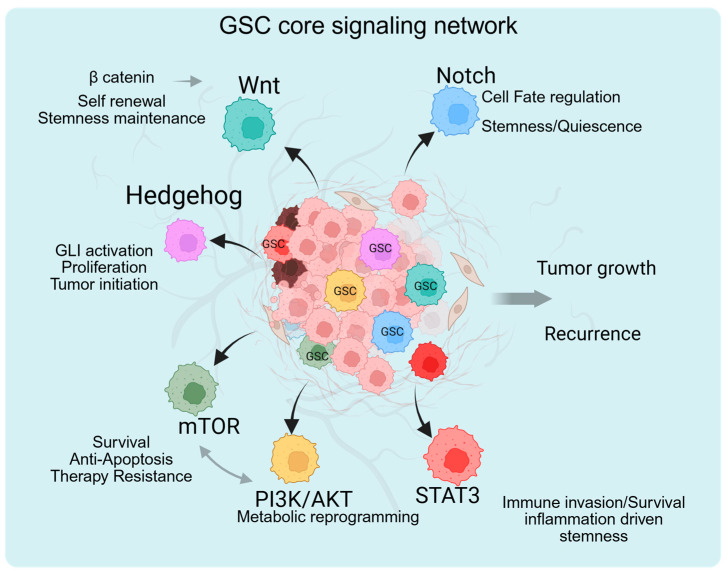
Core signaling networks governing GSC maintenance. Schematic representation of key developmental and oncogenic signaling pathways governing GSC heterogeneity and function within the tumor microenvironment. Central clusters depict distinct GSC states, including proliferative, quiescent, and therapy-resistant subpopulations. Outward arrows represent key pathophysiological outcomes, including tumor growth, invasion, recurrence, and therapeutic resistance. Image created in BioRender.com.

GSCs are supported by a complex, highly interconnected network of developmental and oncogenic signaling pathways (Figure 2 and Table 2). These pathways are aberrantly activated in glioblastoma, promoting self-renewal, proliferation, metabolic adaptation, and therapeutic resistance. Importantly, these cascades do not function in isolation; extensive crosstalk and feedback loops reinforce stemness programs and enable plastic responses to environmental stress.

### 3.1. Developmental Signaling Pathways

Developmental pathways that regulate embryonic neurogenesis are frequently hijacked in GSCs. Notch, Wnt/β-catenin, and Hedgehog signaling pathways play central roles in maintaining stem cell identity and lineage specification during brain development and are aberrantly reactivated in GBM.

#### 3.1.1. Notch Signaling Pathway: Role in Self-Renewal and Cell Fate Determination

The Notch pathway is a highly conserved signaling cascade governing cell fate decisions, stem cell maintenance, and differentiation. Canonical Notch signaling is initiated through ligand–receptor interactions (Jagged or Delta-like ligands binding to Notch receptors), followed by γ-secretase-mediated cleavage and release of the Notch intracellular domain (NICD), which translocates to the nucleus to regulate target genes such as HES and HEY family members. In GSCs, Notch signaling is frequently upregulated and contributes directly to self-renewal and maintenance of an undifferentiated state. Inhibition of Notch signaling via γ-secretase inhibitors reduces sphere formation, decreases stem cell marker expression, and impairs tumor initiation in xenograft models [57]. Notch activation promotes asymmetric division patterns that sustain a stem-like pool while generating differentiated progeny, reinforcing hierarchical organization [13,68]. Furthermore, Notch signaling enhances resistance to radiation and chemotherapy by regulating anti-apoptotic genes and promoting DNA repair mechanisms. Elevated Notch activity correlates with poor prognosis in GBM patients. Crosstalk with hypoxia signaling: The hypoxic tumor microenvironment strongly influences Notch signaling. Hypoxia-inducible factors, particularly HIF-1α, interact with NICD to stabilize and potentiate Notch transcriptional activity. Under low-oxygen conditions, HIF-1α enhances Notch target gene expression, reinforcing stemness and inhibiting differentiation. This cooperative interaction is particularly relevant in perinecrotic hypoxic niches, where GSCs are enriched. Thus, Notch signaling integrates developmental cues with environmental stress responses, sustaining GSC populations in hostile microenvironments.

#### 3.1.2. Wnt Signaling Pathway/β-Catenin: Regulation of Proliferation and Stemness

The canonical Wnt pathway plays a pivotal role in embryonic development and neural stem cell proliferation. The binding of Wnt ligands to Frizzled receptors stabilizes β-catenin, allowing its nuclear translocation and activation of TCF/LEF transcription factors. In GSCs, aberrant Wnt/β-catenin signaling enhances proliferation, promotes self-renewal, and maintains stemness-associated transcriptional programs. β-Catenin directly regulates the expression of oncogenes and stemness genes, including c-MYC, Cyclin D1, SOX2, and NANOG, supporting tumor propagation and resistance to therapy [69,70,71]. Inhibition of Wnt signaling reduces neurosphere formation and impairs tumor growth in preclinical GBM models, highlighting its functional importance in GSC maintenance [72].

Although classical epithelial–mesenchymal transition (EMT) is not strictly observed in glial tumors, GBM exhibits mesenchymal transition-like phenotypes characterized by enhanced invasiveness, therapy resistance, and plasticity. Wnt/β-catenin signaling intersects with EMT-associated transcription factors, including ZEB1, SNAIL, and TWIST, promoting a mesenchymal-like and invasive state. [31,73]. Mesenchymal transition in GBM is closely linked with increased stemness, inflammatory signaling, and adaptive resistance, indicating that Wnt signaling contributes not only to proliferative capacity but also to the aggressive, plastic phenotypes characteristic of GSC subpopulations.

#### 3.1.3. Hedgehog Signaling Pathway

##### GLI Transcription Factors in GSC Maintenance

The Hedgehog (HH) signaling pathway plays a critical role in neural development and stem cell maintenance. The binding of Sonic Hedgehog (SHH) to its receptor PTCH relieves the inhibition of Smoothened (SMO), leading to the activation of GLI transcription factors. In glioblastoma stem cells (GSCs), GLI1 and GLI2 drive the expression of genes associated with cell cycle progression, stemness, and survival, thereby sustaining tumor-propagating capacity [59,74]. Elevated GLI activity correlates with increased tumorigenicity and poor clinical outcomes in GBM patients [74]. Notably, Hedgehog signaling interacts with other developmental pathways, including Notch and Wnt, forming a coordinated regulatory network that reinforces stem-like states [75]. Pharmacologic inhibition of SMO, such as with vismodegib, has shown clinical efficacy in Hedgehog-driven malignancies like basal cell carcinoma [76]. However, targeting Hedgehog signaling in GBM has yielded limited clinical benefit, likely due to pathway redundancy, compensatory activation of GLI transcription factors, and constraints imposed by the blood–brain barrier [77]. As a result, direct inhibition of GLI proteins is being explored as a more effective therapeutic strategy.

### 3.2. Oncogenic and Survival Pathways

Beyond developmental cascades, GSCs depend on aberrantly activated oncogenic signaling networks that promote survival, metabolic adaptation, and therapeutic resistance.

#### 3.2.1. PI3K/AKT/mTOR Signaling Pathway: Frequent Activation in GBM

The PI3K/AKT/mTOR axis is among the most frequently dysregulated pathways in GBM, commonly driven by receptor tyrosine kinase amplification, PTEN loss, or PIK3CA mutations (Cancer Genome Atlas Research Network, 2008) [7]. Activation of this pathway enhances protein synthesis, cell growth, and survival. In GSCs, PI3K/AKT signaling sustains stemness by regulating transcription factors such as SOX2 and other self-renewal-associated genes [56]. mTOR functions as a central integrator of growth factor and nutrient signals, coordinating biosynthetic and metabolic programs essential for tumor propagation. It regulates glycolysis, lipid synthesis, and mitochondrial function, enabling GSCs to adapt to metabolic stress [78]. Hyperactivation of PI3K/AKT/mTOR signaling also contributes to resistance to radiation and temozolomide through anti-apoptotic signaling and enhanced DNA repair mechanisms [79]. Although mTOR inhibitors, including rapamycin analogs (rapalogs), have demonstrated preclinical efficacy, clinical outcomes in GBM have been modest, likely due to feedback activation loops and extensive pathway crosstalk [80].

#### 3.2.2. STAT3 Signaling Pathway: Stemness Transcriptional Program

STAT3, a key transcription factor, dimerizes and translocate to the nucleus upon phosphorylation, then regulates genes involved in proliferation, survival, and stemness. In GSCs, STAT3 drives the expression of core stemness regulators, including SOX2 and c-MYC, as well as anti-apoptotic genes, including BCL-XL, thereby promoting tumor initiation and maintenance [26,81]. Genetic or pharmacologic inhibition of STAT3 reduces sphere formation and tumorigenic potential, underscoring its functional importance in GSC biology [81]. In addition to its intrinsic effects on tumor cells, STAT3 contributes to immune evasion by inducing immunosuppressive cytokine production and upregulating PD-L1 expression [82]. Activation of STAT3 in both tumor cells and tumor-associated macrophages promotes an immunosuppressive microenvironment, favoring stem cell-mediated immune escape mechanisms.

Collectively, these developmental and oncogenic signaling pathways—Hedgehog–GLI, PI3K/AKT/mTOR, and STAT3—underscore the remarkable plasticity and resilience of glioblastoma stem cells (GSCs). Furthermore, incorporation of BBB-penetrant delivery platforms, precision-targeted therapies guided by single-cell and spatial profiling, and combinatorial regimens with epigenetic or metabolic modulators may provide a path toward durable elimination of therapy-resistant GSC populations. In summary, a deeper mechanistic understanding of these interconnected networks, combined with innovative delivery and combination strategies, is essential to overcome GSC adaptive resilience and improve clinical outcomes in glioblastoma.

#### 3.2.3. RTK Amplification (EGFR, PDGFR)

Receptor tyrosine kinase (RTK) amplification represents a defining molecular hallmark of glioblastoma (GBM), contributing to tumor initiation, progression, and therapeutic resistance. Large-scale genomic analyses indicate that aberrations in RTK signaling occur in the majority of GBM cases, with amplification and mutation of the epidermal growth factor receptor (EGFR) being the most prevalent alterations [83]. These alterations frequently coexist with dysregulation of downstream signaling effectors, underscoring the central role of RTK-driven oncogenic networks in GBM pathobiology. Among EGFR alterations, the constitutively active mutant EGFRvIII is particularly significant. This variant arises from an in-frame deletion of exons 2–7, resulting in a truncated receptor that lacks ligand-binding capacity but remains constitutively active [83]. EGFRvIII promotes sustained activation of key downstream pathways, including PI3K/AKT/mTOR, RAS/ERK, and JAK/STAT signaling cascades, thereby enhancing tumor cell proliferation, survival, invasion, and metabolic adaptation [84]. Persistent activation of STAT3, observed in a large proportion of GBM tumors, further contributes to oncogenesis by regulating transcriptional programs associated with cell cycle progression, anti-apoptotic signaling, and immune evasion. Importantly, EGFRvIII-driven signaling differs both quantitatively and qualitatively from wild-type EGFR, reflecting altered receptor trafficking and impaired degradation, which collectively sustain oncogenic signaling outputs [84].

Intratumoral heterogeneity in EGFRvIII expression adds layer of complexity. Distinct subclonal populations within the same tumor may variably express EGFRvIII or other RTK alterations, generating diverse signaling dependencies that promote clonal evolution and therapeutic resistance. This heterogeneity is further compounded by the amplification of additional RTKs, such as PDGFRA, which supports progenitor-like transcriptional states and glioma stem cell (GSC) maintenance. Together, these findings highlight the coexistence of multiple RTK-driven oncogenic programs within individual tumors. A major challenge in targeting RTKs in GBM lies in the extensive crosstalk and redundancy among downstream signaling pathways. RTKs activate a network of interconnected cascades, including PI3K/AKT/mTOR, RAS/MAPK, NF-κB, and STAT3 pathways [85]. Inhibition of a single RTK or pathway often leads to compensatory activation of parallel signaling pathways, thereby limiting therapeutic efficacy. For example, EGFR inhibition can trigger adaptive reactivation of PI3K/AKT or STAT3 signaling, contributing to treatment resistance and tumor persistence [86]. Moreover, genetic alterations such as PIK3CA mutations or PTEN loss can sustain PI3K signaling independently of upstream RTK activity, further diminishing the effectiveness of RTK-targeted monotherapies [85]. Collectively, these observations underscore the importance of signaling plasticity in GBM, where RTK amplification and mutation drive a highly adaptable oncogenic network. The coexistence of heterogeneous RTK alterations and redundant downstream pathways necessitates combinatorial therapeutic strategies that simultaneously target multiple nodes within the signaling network. Future approaches integrating single-cell and spatial transcriptomic analyses will be critical to unravel the dynamic interplay between RTK signaling, tumor heterogeneity, and microenvironmental cues, ultimately enabling the development of more effective, precision-based therapies for GBM.

### 3.3. Epigenetic and Transcriptional Regulation in Glioblastoma Stem Cells

Glioblastoma stem cells (GSCs) exhibit dynamic, highly plastic transcriptional programs that are tightly regulated by epigenetic modifications, non-coding RNAs, and higher-order chromatin architecture. These mechanisms collectively sustain stemness, promote therapy resistance, and enable rapid adaptation to environmental and therapeutic stress. Some of these are summarized in Table 3.

#### 3.3.1. DNA Methylation and Histone Modifications

Aberrant DNA methylation patterns in GSCs contribute to transcriptional dysregulation and lineage plasticity. Promoter hypermethylation of tumor suppressor genes or differentiation-associated loci reinforces stem-like states, while hypomethylation of oncogenic pathways promotes proliferation and survival. MGMT promoter methylation is clinically significant, as it predicts responsiveness to temozolomide chemotherapy; methylated tumors exhibit reduced MGMT expression and increased sensitivity, whereas unmethylated tumors are resistant to TMZ-mediated cytotoxicity [54]. Beyond MGMT, global epigenetic reprogramming orchestrates the expression of key stemness regulators, such as SOX2, OLIG2, and NANOG, enabling GSCs to dynamically shift transcriptional states. Histone modifications further modulate chromatin accessibility and gene expression. Acetylation of histone H3 and H4 correlates with transcriptional activation of stemness and proliferation genes, whereas histone methylation marks, including H3K27me3 and H3K4me3, establish repressive and active chromatin domains, respectively [16]. GSCs leverage these modifications to rapidly adapt transcriptional programs in response to therapeutic stress or microenvironmental cues, including hypoxia and nutrient deprivation. As highlighted by Malta et al. (2024) [92], DNA methylation and broader epigenetic landscapes are not static in gliomas but instead undergo dynamic remodeling from newly diagnosed to recurrent tumors, with distinct evolutionary trajectories depending on IDH1 status and treatment regimen. This reinforces the concept that epigenetic stability and drift may contribute to tumor recurrence, intratumoral heterogeneity, and therapy resistance [92].

#### 3.3.2. Non-Coding RNAs: MicroRNAs and Long Non-Coding RNAs

Non-coding RNAs provide an additional layer of post-transcriptional and epigenetic regulation in GSCs. MicroRNAs (miRNAs) such as miR-21, miR-34a, and miR-128 modulate stemness, proliferation, and apoptosis by targeting differentiation pathways, DNA repair genes, and oncogenic signaling cascades [87,88]. For example, miR-21 enhances chemoresistance by suppressing pro-apoptotic genes, whereas miR-34a acts as a tumor suppressor, promoting differentiation and cell cycle arrest. Long non-coding RNAs (lncRNAs) also play critical roles in GSC maintenance. LncRNAs such as HOTAIR, MALAT1, and NEAT1 interact with chromatin-modifying complexes, including PRC2 and SWI/SNF, to regulate transcriptional networks essential for stemness, proliferation, and therapeutic resistance [89,93]. By modulating enhancer–promoter interactions and recruiting epigenetic modifiers, lncRNAs establish stable yet adaptable transcriptional states that reinforce GSC identity.

#### 3.3.3. Super-Enhancer Networks

Recent studies have highlighted the pivotal role of super-enhancers, large clusters of enhancers densely occupied by transcriptional coactivators such as Mediator and BRD4, in sustaining expression of key stemness genes in GSCs [90]. Super-enhancers orchestrate high-level transcription of lineage-defining transcription factors, including SOX2, OLIG2, and POU3F2, establishing transcriptional dependencies that render GSCs particularly vulnerable to disruption of these regulatory hubs. Pharmacologic targeting of super-enhancer-associated coactivators, such as BET bromodomain inhibitors, has been shown to selectively impair GSC proliferation and tumor propagation, highlighting potential therapeutic vulnerabilities [91].

#### 3.3.4. Integration of Epigenetic Regulation with Therapy Resistance

Epigenetic and transcriptional networks in GSCs intersect with multiple mechanisms of therapy resistance. DNA methylation and histone modifications contribute to quiescence, DNA repair efficiency, and metabolic reprogramming, while non-coding RNAs regulate the expression of ABC transporters, anti-apoptotic proteins, and DNA damage response components [10,16,94]. Super-enhancer-driven transcriptional programs reinforce these survival pathways, integrating intrinsic and extrinsic cues to maintain stemness under therapeutic stress [90,91]. Collectively, these layers of regulation underscore that surface markers alone do not define GSC identity but instead reflect a highly plastic, epigenetically wired cellular state. Disrupting these regulatory networks offers a promising avenue to overcome resistance and selectively target the tumor-propagating GSC population.

## 4. Microenvironmental Niches Supporting GSCs

A microenvironmental niche is a well-defined and specified region surrounding the glioblastoma tumor, which maintains the stemness of the GSCs and aids in tumor growth [95]. The GSC-supportive microenvironment niche is a complex intertwined network comprising an extracellular matrix (ECM), which provides the physical scaffolding by macromolecules such as collagen, fibronectin, hyaluronan and laminin and regulates the stemness via cell signaling pathways [96]. The surrounding cellular components, including endothelial cells, astrocytes, neural cells, microglia, and tumor-associated macrophages (TAM), produce and modify the ECM. Physicochemical components, such as growth factors, chemokines, cytokines, gradients of oxygen and nutrients, modulate ECM and signaling pathways [96,97]. This interlinked regulatory network creates a dynamic niche that sustains tumor aggressiveness, preserves stemness and therapeutic resistance.

### 4.1. Hypoxic Niche

Glioblastoma contains severe hypoxic microregions due to abnormal vasculature and rapid proliferation. These areas form a hypoxic stem cell niche that actively preserves GSCs and is a source of therapy resistance and a major cause of recurrence. The hypoxic necrotic core niche is identified as a unique feature across GBM subtypes [97]. This necrotic core is characterized by a severe region of oxygen and nutrient deprivation. It arises from the tumor’s rapid growth, which alienates it from functional blood vessels and leads to intratumoral thrombosis [32,98,99].

#### 4.1.1. Role of HIF-1α and HIF-2α

Hypoxia-inducible factors (HIFs) are transcription factors that are composed of an oxygen-regulated α subunit (HIF-1α or HIF-2α) and a constitutive β subunit. It regulates the expression of genes involved in aggressive, pro-invasive and highly immunosuppressive phenotypes [97]. HIF-1α is universally expressed and contributes to the expansion and differentiation of the GSC toward an astrocytic lineage mediated via the ERK and PI3K/AKT signaling pathways. HIF1α activation leads to augmentation of Notch signaling through the HIF1α/STAT3 co-activator complex and JAK1/2-STAT3 transcription via VEGF which is essential for GSC population maintenance [100]. HIF2α is preferentially expressed in GSC in moderate hypoxia condition [101]. In GSCs, HIF signaling activates stemness-associated genes, including SOX2, OCT4, and NANOG, thereby reinforcing an undifferentiated phenotype. While both isoforms contribute to hypoxic responses, HIF-2α has been reported to play a particularly important role in sustaining the tumor-initiating capacity of GSCs. Increased HIF activity also enhances expression of angiogenic factors such as VEGF, which further modifies the tumor microenvironment [102]. HIF-2α stimulates the expression of VEGF, which promotes new blood vessel formation. It also promotes invasion of GSCs through the A3 adenosine receptor [100,103].

#### 4.1.2. Hypoxia-Driven Stemness

Experimental studies demonstrate that low oxygen tension increases neurosphere formation, enhances expression of stem cell markers, and expands the population of tumor-propagating cells [104]. Hypoxia can also induce dedifferentiation of more differentiated tumor cells, allowing them to re-acquire stem-like characteristics. This dynamic reprogramming contributes to tumor plasticity and may explain the persistence of GSC populations following therapy [105]. Hypoxia upregulates the expression of stem cell transcription factors like OCT4, SOX2, NANOG, and c-MYC. HIF-2α directly regulates several of these genes. HIF-1α increases expression of Notch ligands (e.g., DLL4). Hypoxia enhances Notch signaling, which maintains an undifferentiated state by preventing glial differentiation and promoting self-renewal in the Wnt/β-catenin pathway. Hypoxia stabilizes β-catenin, which enhances tumor initiation capacity via PI3K/Akt/mTOR signaling and increases metabolic flexibility, sustaining GSC phenotype [32,97].

#### 4.1.3. Metabolic Adaptation

GSCs are metabolically flexible and dynamically adapt to oxygen limitation. Hypoxia drives a strong glycolytic phenotype in GSC [106]. HIF-1α upregulates glycolytic enzymes and glucose transporters, such as Glucose transporter 1 (GLUT1) and Hexokinase 2, allowing tumor cells to generate energy even under low-oxygen conditions. Due to increased expression of GLUT1/GLUT3, glucose uptake also increased. The activity of enzymes like HK2, PFK, and PKM2 leads to accelerated glycolytic flux and upregulates LDHA for the conversion of glucose to lactate. Hypoxia reduces mitochondrial oxidative metabolism by activating enzymes such as pyruvate dehydrogenase kinase 1, which inhibits pyruvate entry into the tricarboxylic acid cycle. This prevents excessive reactive oxygen species formation and protects GSCs from oxidative stress [107,108,109].

Emerging evidence highlights that purinergic signaling and broader purine metabolism are not only critical for sustaining tumor growth but also contribute to glioblastoma progression, including glioblastoma stem-like cell (GSC) invasion and therapeutic response. In particular, adenosine signaling through the A3 adenosine receptor (A3AR) has been implicated in promoting GSC invasive behavior and tumor aggressiveness, linking extracellular adenosine dynamics to malignant phenotypes in glioblastoma. In parallel, metabolic rewiring of purine pathways has been shown to influence treatment sensitivity, including response to temozolomide. As demonstrated by D’Aprile et al. (2025), modulation of purine metabolic pathways significantly enhances glioblastoma susceptibility to temozolomide treatment, underscoring the functional relevance of purine metabolism in therapeutic resistance and response regulation [110].

### 4.2. Perivascular Niche

The perivascular niche (PVN) is the specific microenvironment that is adjacent to the brain vasculature. It is mainly composed of endothelial Cells, astrocytes, pericytes, tumor cells, immune cells, and ECM. Here, the GSCs are directly in physical interaction with endothelial cells (ECs) through the vascular structures such as capillaries and arterioles. This interaction leads to GSC survival via the expression of various stemness genes [111,112,113].

#### 4.2.1. Interaction with Endothelial Cells

In GBM, ECs contact GSCs, which activates the EC and induces an invasive phenotype. The ECs’ angiopoietin (Ang-1) and Tie 2 receptors on GSCs upregulate adhesion proteins like N-cadherin and integrin β1 and facilitate GSC invasion. Interaction between ECs and GSCs promotes angiogenesis by releasing pro-angiogenic factors (e.g., VEGF, FGF-2, PDGF), which upregulate the expression of Bmi-1, Sox-2, Olig-2, and Shh, thereby maintaining the stemness and expansion of the vascular niche. Thus, GSC can promote angiogenesis either directly via VEGF secretion and differentiation into endothelial cells, or indirectly by inducing endothelial cells to secrete angiocrine factors [113,114].

#### 4.2.2. Angiocrine Signaling

Major angiocrine signaling involves self-renewal and maintenance of stemness, making it the Notch Signaling Pathway [114]. This signaling helps GSCs to generate endothelial-like cells that contribute to tumor angiogenesis. Endothelial cells express high levels of Notch ligands Jagged-1 (JAG-1) and Delta-like ligand 1 and 4 (DLL4) that activate Notch receptors 1 and 2 on GSCs and promote self-renewal. Binding of Notch ligands and receptors causes the Notch intracellular domain (NICD) to be released into the nucleus, where it induces the transcription of Notch target genes: Hairy Enhancer of Split 1 (HES1), HES-related proteins (HEY), p21 (CDKN1A), Cyclin D1 (CCDN1), c-myc (MYC), BCL2, DTK1 (Deltex1) and NF-κB2. This activation sustains the stemness, self-renewal, and therapeutic resistance of the GSC. Additionally, paracrine factors secreted from ECs in the vascular niche activate the mTOR pathway and promote the expansion of GSCs [97,115]. Emerging evidence suggests that developmental signaling pathways intersect with redox-regulatory mechanisms to control neural stem cell (NSC) fate decisions. In particular, a NOTCH–NRF2 axis has been described in NSCs, linking NOTCH-mediated stem cell maintenance programs with NRF2-dependent oxidative stress responses and metabolic regulation. Under neuroinflammatory and oxidative stress conditions, NRF2 activation plays a key role in preserving NSC viability and regulating differentiation outcomes in coordination with NOTCH signaling. Given the proposed developmental and functional relationship between NSCs and glioblastoma stem-like cells (GSCs), this axis may represent an important mechanism contributing to GSC maintenance and therapy resistance by integrating niche-derived stress signals with intrinsic stemness pathways. Recent work by Torrisi et al. further supports this concept, demonstrating that NSC fate under neuroinflammatory conditions is tightly coupled to oxidative stress response programs involving NRF2 signaling [116]. Incorporating this NOTCH–NRF2 interplay provides a more comprehensive framework for understanding how developmental pathways converge with metabolic and redox regulation in GSC biology.

#### 4.2.3. Extracellular Matrix (ECM) Regulation

In the perivascular niche, GSCs interact with ECM components such as laminin, collagen IV, and fibronectin. GSCs and ECs secrete enzymes like Matrix Metalloproteinase MMP-2, MMP-9, Urokinase-type plasminogen activator (Upa) that degrade the ECM components and remodel ECM. GSC bind to laminin-rich vascular basement membrane through integrin α6β1 via laminin α2 for GSC maintainance. HIF-1α also supports GSC survival by promoting ECM modeling and angiogenesis [112,113].

#### 4.2.4. Vascular Mimicry

Tumor cells, particularly GSC, form vessel-like channels, perfusable structures like an endothelial network but without true endothelial cells [117]. This phenomenon allows tumors to bypass anti-angiogenic therapy and maintain nutrient supply, serving as an alternative to angiogenesis for survival. Vascular mimicry is characterized by the presence of markers like vascular endothelial (VE)–cadherin, Matrix Metalloproteinase (MMP)-2, EphA2, membrane type 1 (MT1)-MMP (MMP-14), and laminin. Embryonic and stem cell signaling are responsible for the characteristics of plasticity associated with vascular mimicry [117]. The hypoxic tumor microenvironment promotes vascular mimicry and GSC maintenance.

### 4.3. Immune Microenvironment

The immune microenvironment of glioblastoma (GBM) is highly immunosuppressive and plays a crucial role in maintaining GSC [118]. Rather than eliminating tumor cells, the tumor often reprograms immune components to promote stemness, survival, and therapy resistance. It comprises various immune cells including tumor-associated macrophages (TAMs), myeloid-derived suppressor cells (MDSCs) and other immune cells, tumor cells and a cytokine network [119].

#### 4.3.1. Tumor-Associated Macrophages (TAMs)

TAMs are the most abundant immune cells in GBM and may constitute 30–50% of the tumor mass [120]. They originate from brain-resident microglia infiltrating monocyte-derived macrophages. Within the tumor, these cells are reprogrammed toward an M2-like immunosuppressive phenotype. GSCs actively recruit macrophages by secreting chemokines such as CCL2 (MCP-1) CSF-1 CXCL12 VEGF. These signals attract circulating monocytes and promote their differentiation into TAMs within the tumor microenvironment. These TAMs suppress cytotoxic immune responses and support tumor progression [121]. TAMs secrete several growth factors (EGF, TGF-β, PDGF, IL-6, IL-10, MMPs that promote GSC maintenance [119,122].

#### 4.3.2. Immune Checkpoint Expression (PD-L1)

A major immune escape mechanism in GSC survival involves high expression of immune checkpoint ligands. The most important is the PD-1/PD-L1 axis. PD-1 receptor on T cells binds to PD-L1 ligand on GSC and immune cells which suppresses T-cell activation and decreased cytokine production. Expression of programmed death-ligand 1 (PD-L1) on GSCs suppresses T-cell activation by engaging PD-1 receptors on immune cells [123]. Hypoxia and inflammatory signaling can further increase PD-L1 expression, reinforcing immune suppression within the tumor microenvironment. Elevated PD-L1 levels have been associated with reduced immune infiltration and poor clinical outcomes in glioblastoma [124,125].

#### 4.3.3. Immune Checkpoint Evasion

Glioblastoma stem cells (GSCs) utilize a varied array of immune evasion strategies that help them in sustaining stemness, enabling escape from immune surveillance, tumor progression and recurrence [126]. Secretion of immunosuppressive cytokines, including transforming growth factor-β (TGF-β), interleukin-10 (IL-10), and prostaglandin E2 (PGE2), is the central mechanism that collectively shifts the tumor microenvironment toward an immunotolerant state and suppresses effective antitumor immune responses. In parallel, GSCs promote the recruitment and expansion of regulatory T cells (Tregs), which inhibit the activity of effector T cells, antigen-presenting cells (APCs), and natural killer (NK) cells, thus further reducing immune activation. Another important mechanism is the downregulation of major histocompatibility complex class I (MHC-I) molecules on tumor cells, which weakens antigen presentation and inhibits recognition by cytotoxic T lymphocytes. Additionally, chronic antigen exposure within the tumor microenvironment, coupled with activation of immune checkpoint pathways, drives T-cell exhaustion, characterized by reduced proliferative capacity and weakened effector function, ultimately weakening antitumor immunity. Importantly, GSCs also engage in dynamic crosstalk with tumor-associated macrophages (TAMs), establishing a reciprocal relationship in which GSCs promote TAM recruitment and polarization toward an immunosuppressive phenotype, while TAMs secrete factors that reinforce GSC maintenance and stem-like properties. Together, these organized mechanisms create a highly immunosuppressive niche that supports tumor continuity, highlighting the need for therapeutic strategies that concurrently target both GSCs and the immune microenvironment [122,125,127,128].

### 4.4. Extracellular Matrix and Mechanical Cues

In GBM, the ECM and mechanical properties of the tumor microenvironment play a critical role in maintaining GSCs. ECM remodeling by tumor cells and stromal cells creates a microenvironment favorable for GSC expansion. The transmembrane receptors integrins bind ECM ligands, and intracellular signaling pathways (PI3K/Akt, MAPK/ERK pathways) are activated. Integrin α6 is frequently enriched in GSC populations and is considered a marker associated with aggressive tumor behavior. Focal adhesions are multiprotein complexes (FAK, Talin, Paxillin, Vinculin) that connect the ECM to integrins and help organize the Cytoskeleton. GBM cells actively modify the ECM by secreting enzymes such as Matrix Metalloproteinases (MMPs) and Cathepsins. These enzymes degrade ECM components and stimulate integrin signaling and stemness [129,130]. Normal brain tissue is relatively soft. However, GBM tumors exhibit increased stiffness due to ECM accumulation, collagen deposition, cellular density and interstitial pressure. In GBM, increased matrix stiffness activates mechanosensitive pathways, including FAK, Rho/ROCK signaling, and YAP/TAZ transcriptional regulators, via Piezo1, a mechanosensitive ion channel, the PI3K/Akt pathway, and CD40/NF-κB2. These pathways regulate gene expression associated with stemness. Mechanical signals transmitted through integrins lead to cytoskeletal rearrangements, enhancing GSC adhesion to ECM [131].

## 5. Emerging Therapeutic Strategies Targeting GSCs

**Figure 3:**
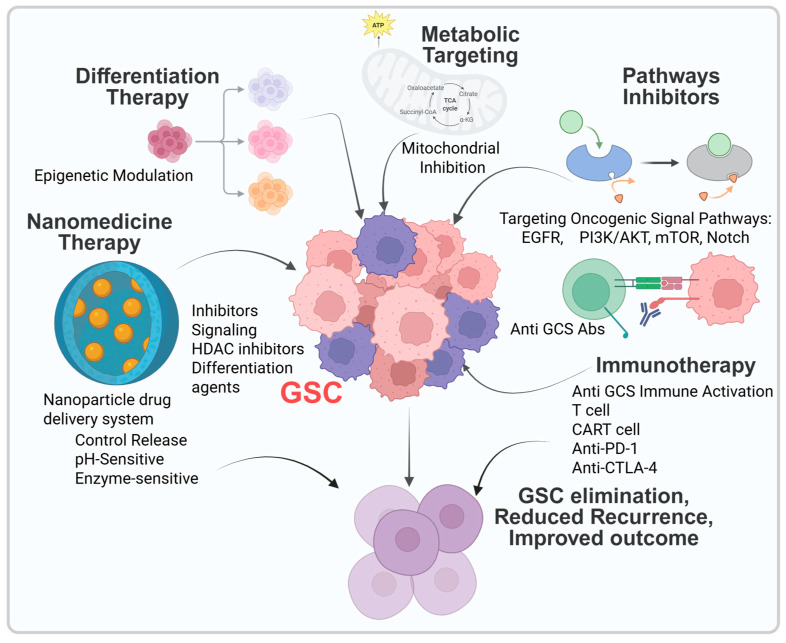
Therapeutic targeting strategies for glioma stem cells (GSCs) in glioblastoma. Schematic overview of multi-pronged therapeutic strategies targeting glioblastoma stem cells (GSCs) to improve treatment outcomes. The illustration summarizes major intervention approaches, including differentiation therapy, epigenetic modulation (DNA methylation/demethylation and histone acetylation), mitochondrial inhibition and inhibition of oncogenic signaling pathways such as EGFR, PI3K/AKT/mTOR and Notch. Nanomedicine-based drug delivery systems are shown to enable controlled, pH- and enzyme-responsive release of therapeutic agents, including signaling inhibitors and differentiation-inducing compounds. Immunotherapy strategies targeting GSCs include activating T cells and CAR-T cells, as well as immune checkpoint blockade (anti–PD-1 and anti–CTLA-4), and antibody-based targeting of GSC-associated antigens. Image was created in BioRender.com.

Glioblastoma stem cells (GSCs) play a crucial role in tumor initiation, recurrence, and therapeutic resistance in glioblastoma (GBM). Subsequently, multiple therapeutic strategies have been developed to selectively target or reprogram this stem-like compartment, including pathway-specific inhibitors, differentiation therapies, immunotherapies, metabolic targeting, and nanotechnology-based delivery systems (Figure 3 and Table 4). Unfortunately, clinical translation remains challenging due to factors including but not limited to signaling redundancy, intratumoral heterogeneity, and limited drug penetration across the blood–brain barrier (BBB) [16,26,132]. Here, targeting glioblastoma stem cells represents one of the most promising yet challenging strategies for improving GBM outcomes. While preclinical studies have demonstrated that GSCs can be realistically targeted through these pathway disruptions, translation into clinical success has been limited. A central challenge lies in the extensive redundancy and crosstalk among signaling pathways, including Notch, Wnt, Hedgehog, PI3K/AKT, and STAT3. Inhibition of a single pathway often leads to compensatory activation of parallel signaling cascades, thereby sustaining stemness and survival. Recent advances have also highlighted neural stem cell-based approaches as emerging and innovative platforms for glioblastoma treatment. These strategies exploit the intrinsic tumor-tropic properties of neural stem cells for targeted delivery of therapeutic agents and modulation of the tumor microenvironment, offering a complementary avenue to existing GSC-directed therapies [133].

Another critical obstacle is intratumoral heterogeneity and plasticity. GSCs exist in multiple transcriptional and metabolic states, including proneural, mesenchymal, and quiescent subtypes, which can dynamically interconvert in response to therapeutic pressure [17]. This plasticity enables tumor adjustment and promotes recurrence, highlighting the need for multi-targeted or combinatorial therapeutic strategies rather than single-agent approaches. The tumor microenvironment (TME) further complicates therapeutic targeting. Hypoxia, immune suppression, and interactions with stromal and immune cells reinforce GSC maintenance and therapy resistance. For instance, hypoxia-driven HIF signaling enhances Notch activity and metabolic reprogramming, while STAT3-mediated cytokine signaling promotes immune evasion. Thus, effective therapies must concurrently address cell-intrinsic and microenvironmental mechanisms.

A major translational bottleneck is the blood–brain barrier, which restricts drug delivery to tumor sites. Emerging nanomedicine-based approaches offer promising solutions by improving CNS penetration and enabling targeted delivery to GSC populations. However, challenges related to scalability, safety, and heterogeneity of target expression remain. Immunotherapy has shown transformative success in other malignancies but has been less effective in GBM. The immunologically “cold” tumor environment, low neoantigen burden, and presence of immunosuppressive cells limit responses. Future strategies will likely require combination approaches integrating immunotherapy with pathway inhibitors, oncolytic viruses, or metabolic targeting to enhance immune activation. Overall, the future of GSC-targeted therapy lies in rational combination strategies, patient stratification based on molecular and single-cell profiling, and the integration of advanced delivery systems. Leveraging multi-omics and spatial transcriptomics to map GSC niches and vulnerabilities may further enable the development of precision therapies. Ultimately, overcoming the interconnected challenges of redundancy, plasticity, and delivery will be essential to translate promising preclinical findings into meaningful clinical benefit for patients with GBM.

### 5.1. Targeting Developmental and Oncogenic Pathways

Aberrant activation of developmental pathways such as Notch, Wnt, and Hedgehog, along with oncogenic signaling cascades including PI3K/AKT/mTOR and STAT3, sustains GSC self-renewal and survival.

#### 5.1.1. Notch Pathway (γ-Secretase Inhibitors)

Pharmacologic inhibition of Notch signaling using γ-secretase inhibitors (GSIs) blocks cleavage of the Notch intracellular domain (NICD), thereby suppressing downstream transcriptional programs. GSIs reduces neurosphere formation, decrease stemness marker expression, and enhances radiosensitivity in GSCs [57,58]. However, clinical translation has been limited by gastrointestinal toxicity and compensatory activation of parallel pathways [47]. Combination strategies with radiotherapy or PI3K inhibitors are under active investigation.

#### 5.1.2. Wnt/β-Catenin Pathway

Wnt signaling promotes GSC proliferation and stemness via β-catenin-mediated transcription of oncogenic targets such as c-MYC and Cyclin D1 [31,148,149]. Inhibitors targeting porcupine, tankyrase, or β-catenin–TCF interactions reduce GSC self-renewal in preclinical models. However, pathway redundancy and systemic toxicity due to Wnt’s role in normal tissue homeostasis remain key limitations.

#### 5.1.3. Hedgehog Pathway

Hedgehog signaling regulates stemness through GLI transcription factors. SMO inhibitors such as vismodegib suppress GSC maintenance in preclinical studies [60]. However, clinical efficacy in GBM has been limited due to poor BBB penetration and SMO-independent GLI activation, prompting development of direct GLI inhibitors.

#### 5.1.4. PI3K/AKT/mTOR Pathway

This pathway is frequently activated in GBM due to PTEN loss and receptor tyrosine kinase amplification. mTOR inhibitors (e.g., everolimus, temsirolimus) suppress GSC proliferation and survival [63,150]. However, feedback activation loops and metabolic compensation limit their clinical efficacy, necessitating combination approaches (e.g., PI3K + MEK or STAT3 inhibition).

#### 5.1.5. STAT3 Pathway

STAT3 acts as a central node integrating stemness, survival, and immune evasion. Inhibition of STAT3 using small molecules such as WP1066 reduces tumor growth and GSC proliferation. [151,152,153,154]. Importantly, STAT3 blockade may also enhance antitumor immunity, supporting combination with immunotherapies.

### 5.2. Differentiation Therapy

Differentiation-based strategies aim to convert GSCs into non-proliferative, terminally differentiated cells, thereby reducing their self-renewal capacity and tumor-initiating potential. BMP signaling via BMP4 promotes astrocytic differentiation and suppresses tumorigenicity in glioblastoma stem cells (GSCs), although resistance can arise through epigenetic silence or downregulation of BMP receptors [134]. Retinoic acid (ATRA) similarly induces differentiation and reduces stem-like properties, but its therapeutic impact in glioblastoma has been modest, in part due to limited central nervous system penetration and tumor heterogeneity [134,155]. In parallel, epigenetic modulators such as histone deacetylase (HDAC) inhibitors (e.g., vorinostat) and bromodomain and extra-terminal (BET) inhibitors can reprogram oncogenic transcriptional networks and sensitize tumors to therapy, underscoring the importance of chromatin plasticity in maintaining the GSC state [16,91].

From a broader perspective, these differentiation and epigenetic-based strategies reflect a critical shift in glioblastoma (GBM) therapeutics—from cytotoxic approaches toward reprogramming tumor cell identity. However, clinical translation remains challenging. Recent studies have demonstrated that modulation of key developmental transcription factors and epigenetic regulators can promote neuronal or astrocytic-like differentiation in glioblastoma cells, leading to suppression of proliferation and tumorigenicity [17,156,157]. For instance, forced expression of proneural lineage programs or inhibition of stemness-associated epigenetic circuits has been shown to reduce GSC self-renewal and impair tumor propagation in preclinical models [16]. However, the clinical efficacy of differentiation therapy remains limited due to tumor heterogeneity and reversible cellular plasticity, which allow subsets of GSCs to escape terminal differentiation and re-acquire stem-like properties under microenvironmental pressure. A critical next step in GBM research is expected to involve the precise targeting of context-specific vulnerabilities of GSCs, including the integration of epigenetic therapies with niche-disrupting or immune-modulating strategies, and the use of single-cell and spatial profiling approaches to enable patient-specific therapeutic interventions.

### 5.3. Immunotherapeutic Strategies

Chimeric antigen receptor (CAR)-T cell therapies directed against GSC-associated antigens such as EGFRvIII, IL13Rα2, and HER2 have demonstrated feasibility and safety in early-phase clinical trials, with evidence of transient tumor regression in some patients [136]. However, durable responses remain limited due to pronounced antigen heterogeneity, antigen loss, and a profoundly immunosuppressive tumor microenvironment. Similarly, autologous dendritic cell (DC) vaccines pulsed with tumor lysates have been shown to elicit adaptive immune responses and confer survival benefit in selected patient subsets, highlighting the potential of personalized immunotherapy approaches [137]. Immune checkpoint blockade targeting PD-1/PD-L1 has, in contrast, demonstrated limited efficacy in glioblastoma, largely due to the low tumor mutational burden and an immunologically “cold” microenvironment that restricts T-cell infiltration and activation [138]. Oncolytic viral therapies, including herpes simplex virus (HSV)-based and adenoviral platforms, offer a complementary strategy by directly lysing tumor cells while simultaneously stimulating antitumor immune responses, with emerging evidence of activity against GSC populations in both preclinical and early clinical settings [158,159].

From a broader perspective, these immunotherapeutic approaches underscore both the promise and the current limitations of immune-based strategies in glioblastoma (GBM). While each modality has shown signals of activity, none have yet achieved consistent, durable clinical benefit as monotherapy. A central challenge lies in the dynamic interplay between GSC plasticity and the immunosuppressive niche, which together facilitate immune evasion and therapeutic resistance. Ongoing clinical efforts are therefore increasingly focused on rational combination strategies—such as CAR-T cells combined with checkpoint blockade, oncolytic viruses with immune-priming effects, or vaccines combined with adjuvant immunomodulators—to overcome these barriers. In my view, a critical next step will be integrating immunotherapy with approaches targeting GSC-specific vulnerabilities, including metabolic and epigenetic dependencies, as well as strategies that remodel the tumor microenvironment to enhance immune infiltration and persistence. Advances in single-cell and spatial profiling are likely to play a pivotal role in identifying actionable immune niches and guiding more precise, patient-tailored immunotherapeutic interventions in GBM.

### 5.4. Metabolic Targeting

Glioblastoma stem cells (GSCs) exhibit remarkable metabolic plasticity, enabling survival under fluctuating oxygen and nutrient conditions. This adaptability represents a promising therapeutic vulnerability.

#### 5.4.1. OXPHOS Inhibitors

Some GSC subsets preferentially rely on mitochondrial oxidative phosphorylation (OXPHOS) for energy production and redox homeostasis. Pharmacologic inhibition of mitochondrial respiration using agents such as metformin, phenformin, or IACS-010759 reduces ATP production, induces apoptosis, and impairs stemness properties in preclinical models [19,50,160]. OXPHOS inhibition can also sensitize GSCs to radiotherapy by impairing antioxidant defenses and increasing ROS accumulation.

#### 5.4.2. Glycolysis Inhibitors

Hypoxia-adapted GSCs often rely on aerobic glycolysis (Warburg effect) to sustain proliferation and survival. Inhibition of key glycolytic enzymes, such as Hexokinase 2 (HK2) and lactate dehydrogenase A (LDHA), or glucose transporters reduce proliferation and stem-like features [108,161]. However, systemic toxicity and metabolic compensation through alternative substrates remain major hurdles.

#### 5.4.3. Targeting Redox Balance

GSCs maintain elevated antioxidant capacity, including high levels of glutathione and NADPH, to buffer reactive oxygen species (ROS) generated during therapy [110,162]. Pharmacologic inhibition of glutathione synthesis (e.g., buthionine sulfoximine) or NADPH production sensitizes GSCs to both chemotherapy and radiotherapy, tipping the redox balance toward apoptosis. Combination strategies integrating redox modulators with standard-of-care therapies are being actively explored.

### 5.5. Nanomedicine-Based Drug Delivery

Nanoparticle-based delivery systems have emerged as a promising strategy to overcome the blood–brain barrier (BBB) imposed limitations and improve therapeutic targeting of glioblastoma stem cells (GSCs). Nanoparticles functionalized with ligands such as transferrin, angiopep-2, or receptor-targeting peptides can exploit receptor-mediated transcytosis to enable BBB penetration. In addition, lipid-based and polymeric nanoparticles enhance drug pharmacokinetics, prolong systemic circulation, and protect labile therapeutics from degradation, thereby improving bioavailability within the tumor microenvironment [144,163]. Liposomal and polymeric systems, including clinically used formulations such as liposomal doxorubicin, enable controlled drug release, reduce systemic toxicity, and promote tumor accumulation. These platforms are particularly advantageous for co-delivery strategies, enabling the simultaneous delivery of chemotherapeutics, targeted inhibitors, or nucleic acids to achieve synergistic anti-GSC effects [145,164].

Targeted delivery approaches further refine this strategy by conjugating nanoparticles to antibodies or aptamers targeting stem cell markers, such as CD133, thereby enabling preferential accumulation within stem-like tumor populations [165]. However, the heterogeneity and dynamic regulation of CD133 expression present significant challenges to consistent targeting efficacy across patients and tumor states. To address these limitations, next-generation combination nanotherapeutics are being developed that incorporate multifunctional platforms capable of delivering siRNA, small-molecule inhibitors, and immunomodulatory agents concurrently. These systems aim to simultaneously disrupt oncogenic signaling, metabolic adaptability, and immune evasion mechanisms in GSCs, thereby overcoming compensatory resistance pathways and enhancing therapeutic efficacy [108,163].

#### 5.5.1. BBB-Penetrating Nanoparticles

Nanoparticles functionalized with ligands such as transferrin, angiopep-2, or receptor-targeting peptides can cross the BBB via receptor-mediated transcytosis. Lipid-based and polymeric nanoparticles improve drug pharmacokinetics, prolong circulation, and protect labile therapeutics from degradation [144,163].

#### 5.5.2. Liposomal and Polymeric Systems

Liposomal formulations (e.g., liposomal doxorubicin) and polymeric micelles enable controlled drug release, reduced systemic toxicity, and enhanced tumor accumulation. These systems can co-deliver multiple agents, including chemotherapeutics, small-molecule inhibitors, or nucleic acids, to achieve synergistic anti-GSC effects [164,166,167,168].

#### 5.5.3. Targeted Delivery to CD133^+^ Cells

Antibody- or aptamer-conjugated nanoparticles targeting CD133 selectively enrich drug delivery to stem-like populations [165]. Despite promising preclinical results, the heterogeneity of CD133 expression and dynamic stemness states limits universal efficacy.

#### 5.5.4. Combination Nanotherapeutics

Multifunctional nanoparticles capable of delivering siRNA, small-molecule inhibitors, or immunomodulatory agents simultaneously offer the potential to disrupt signaling pathways, metabolic networks, and immune evasion mechanisms in GSCs. Such platforms are being developed to overcome compensatory resistance and improve therapeutic index [169,170,171,172]. Nanoparticle-based strategies represent a critical advancement in GBM therapeutics by addressing one of the field’s most persistent barriers: effective drug delivery to the brain, particularly to GSC niches. Despite encouraging preclinical progress, clinical translation has been slower than anticipated, largely due to challenges related to delivery efficiency, off-target accumulation, immunogenicity, and scalability. Ongoing efforts are increasingly focused on integrating nanotechnology with precision-targeting approaches, including ligand-directed delivery, stimuli-responsive release systems, and combination regimens with immunotherapy or gene-editing tools. The future of nanotherapeutics in glioblastoma will depend on the ability to tailor delivery systems to tumor-specific and patient-specific contexts, leveraging advances in single-cell and spatial profiling to identify optimal targets and delivery routes. Such integrative strategies hold promise for overcoming BBB constraints and effectively eliminating therapy-resistant GSC populations. The blood–brain barrier (BBB) and intratumor heterogeneity significantly prevent effective delivery of therapeutics to GSCs. Notably, recent work from the Debabrata Mukhopadhyay laboratory has advanced tumor-targeted nanoparticle systems for nucleic acid delivery, including siRNA-based approaches designed to modulate DNA damage response and enhance therapeutic sensitivity [173]. Complementing these advances, a recent study reported the development of surface-engineered, dual-drug-loaded, tumor-targeted liposomal nanoparticles capable of overcoming therapeutic resistance in glioblastoma multiforme [174]. This platform enabled efficient co-delivery of therapeutic agents, improved tumor-specific accumulation, and enhanced blood–brain barrier penetration, leading to reduced tumor burden and prolonged survival in preclinical models. Importantly, the study demonstrated modulation of resistance-associated pathways and improved apoptotic responses, supporting the utility of combinatorial nanotherapeutic strategies in targeting aggressive and stem-like tumor populations [174].

## 6. Challenges in Translating GSC-Targeted Therapies

Despite substantial progress in identifying glioblastoma stem cell (GSC)-specific vulnerabilities, the successful clinical translation of GSC-targeted therapies remains limited. Multiple biological and clinical barriers collectively undermine therapeutic efficacy and durability, underscoring the need for more refined, integrative strategies.

### 6.1. Intratumoral Heterogeneity and Cellular Plasticity

A central challenge in targeting GSCs is their profound intratumoral heterogeneity and phenotypic plasticity. Single-cell transcriptomic studies have demonstrated that GBM tumors harbor diverse GSC subpopulations that recapitulate distinct developmental lineages, including proneural, mesenchymal, and classical states. Importantly, GSCs exhibit dynamic interconversion with non-stem tumor cells, driven by microenvironmental cues such as hypoxia and inflammatory signaling. This bidirectional plasticity enables tumor cells to acquire stem-like properties following therapeutic insult, thereby sustaining tumor propagation and recurrence. Consequently, therapies directed against a single GSC marker or pathway are unlikely to achieve durable responses, as alternative stem-like subclones can persist or emerge [17,49]. Recent advances in multi-omics approaches, particularly single-cell and spatial transcriptomics, have enabled high-resolution characterization of GSC subpopulations and their dynamic interactions with the tumor microenvironment [17,156,175,176,177]. These studies have revealed distinct cellular states, lineage plasticity, and spatially organized tumor niches that cannot be captured by conventional bulk analyses. Importantly, integrative multi-omics analyses have identified functionally and metabolically distinct GSC subsets, including hypoxia-adapted and immunosuppressive populations associated with poor clinical outcomes.

Such heterogeneity has direct therapeutic implications, as it contributes to differential treatment response and limits the efficacy of conventional as well as emerging therapies, including nanoparticle-based delivery systems, which may fail to uniformly target diverse GSC populations. Similarly, therapeutic strategies such as differentiation therapy may be effective only in specific cellular states, further emphasizing the need for precise molecular stratification. Therefore, incorporating omics-driven stratification and functional profiling of GSCs is essential for identifying actionable vulnerabilities across heterogeneous tumor states. Overall, integrating multi-omics data with therapeutic design will be critical for the development of precision strategies capable of targeting diverse GSC populations, overcoming treatment resistance, and improving clinical outcomes in glioblastoma.

### 6.2. Adaptive Resistance Mechanisms

Adaptive resistance further reduces the long-term efficacy of GSC-targeted therapies. Inhibition of key oncogenic pathways often generates compensatory activation of parallel signaling networks, including PI3K/AKT, MAPK, and STAT3 pathways, thereby preserving cell survival and proliferation. In addition to signaling redundancy, GSCs exhibit remarkable metabolic flexibility, shifting between glycolysis and oxidative phosphorylation in response to environmental conditions and therapeutic pressure. This metabolic adaptation favors survival under stress conditions and contributes to resistance against targeted and cytotoxic therapies. These findings underscore the necessity for rational combination strategies that concurrently target multiple signaling and metabolic dependencies [19,26,55].

### 6.3. Blood–Brain Barrier Constraints

The blood–brain barrier (BBB) is not uniformly intact in glioblastoma (GBM), as high-grade tumors often exhibit regions of BBB disruption [178]. However, this disruption is highly heterogeneous both spatially and temporally, resulting in the co-existence of leaky and relatively intact tumor compartments. Consequently, drug delivery is uneven, and infiltrative tumor margins—where glioblastoma stem cells (GSCs) are frequently enriched—often remain protected from adequate therapeutic exposure. In addition, even in regions with compromised BBB integrity, multiple non-BBB-related barriers contribute to therapeutic failure, including elevated interstitial fluid pressure, abnormal tumor vasculature, dense extracellular matrix, and limited intratumoral penetration and retention of therapeutic agents. Furthermore, many targeted drugs and nanoparticle-based systems face additional limitations such as suboptimal tissue diffusion, rapid clearance, and efflux transporter activity (e.g., P-glycoprotein), collectively reducing effective intratumoral drug concentrations. Therefore, current strategies aimed at improving GBM therapy include not only nanoparticle-based delivery systems and convection-enhanced delivery but also approaches that enhance tumor penetration, microenvironmental remodeling, and spatially targeted drug distribution [179,180].

### 6.4. Toxicity to Normal Neural Stem Cells

The overlap between signaling pathways poses a critical limitation for GSC-targeted approaches, which regulate GSC maintenance and normal neural stem cell (NSC) function. Developmental pathways including Notch, Wnt/β-catenin, and Hedgehog play essential roles in neurogenesis, tissue homeostasis, and repair. Systemic inhibition of these pathways may consequently result in substantial neurotoxicity, impair cognitive function, and disrupt normal brain physiology. This challenge imposes the development of more selective targeting strategies that exploit tumor-specific dependencies or delivery systems that favorably target malignant cells while sparing normal NSCs [47,181].

### 6.5. Clinical Trial Design and Biomarker Limitations

Furthermore, challenges in trial design and patient stratification limit clinical translation. GBM displays extensive interpatient and intratumoral molecular heterogeneity, thwarting the identification of common therapies. Additionally, the lack of robust predictive biomarkers limits the ability to select patients most likely to benefit from specific targeted interventions. Traditional clinical trial designs may not sufficiently account for this complexity, resulting in high failure rates. A combination of molecular stratification, adaptive trial designs, and real-time biomarker assessment, potentially guided by single-cell and spatial profiling technologies, may advance therapeutic outcomes and accelerate the development of effective GSC-targeted therapies [138,182,183,184].

## 7. Perspective and Future Directions

Overall, the above-discussed challenges reflect an incomplete understanding of GBM as a dynamic and adaptive system, which has led to the failure of GSC-targeted therapies. Hence, GSCs should be considered as a transient, state-dependent phenotype that develops under specific microenvironmental and therapeutic pressures. This paradigm shift has important therapeutic implications.

**Figure 4:**
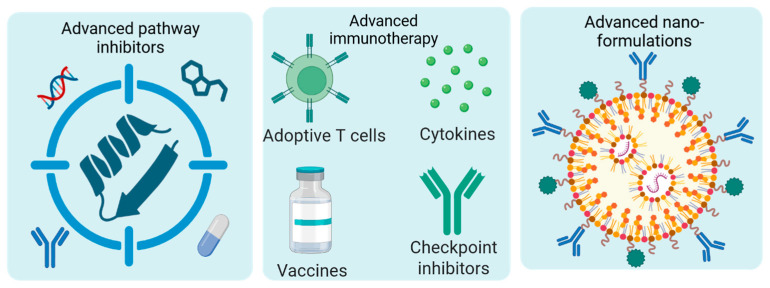
Pathway inhibitors, advanced immunotherapies, and nano-formulations disrupt GSC signaling, cross the BBB, and enable delivery of CAR-T and targeted therapies. The figure was created in BioRender.com.

First, future aims should emphasize the transition from single-pathway inhibition to network-level targeting. Combinative methodologies that concurrently interrupt multiple adaptive intersections are undoubtedly required to achieve lasting responses (Figure 4). For example, combining RTK inhibition with metabolic targeting or epigenetic modulators may prevent compensatory effects and minimize tumor malleability. Second, since the tumor microenvironments differentially regulate GSC states, therapies that disrupt these niche-specific signals, such as vascular normalization strategies or modulation of tumor-associated immune cells, may synergize with direct GSC targeting. Third, incorporating single-cell and spatial multi-omics technologies will offer unique opportunities to resolve intratumoral heterogeneity and incorporate context-specific susceptibilities into clinical trial design, enabling real-time patient stratification and adaptive therapeutic interventions, thereby improving trial success rates. Finally, selective targeting remains an important priority. The overlap between GSC and normal NSC regulatory pathways calls for the identification of tumor-specific dependences, such as synthetic lethal interactions or lineage-restricted vulnerabilities. Further, precision delivery platforms, including nanoparticle-based systems or ligand-directed therapies, may enhance tumor specificity while minimizing off-target toxicity.

## 8. Conclusions

Over the past two decades, a growing body of preclinical and clinical data has established that glioblastoma stem cells (GSCs) represent a critical cellular reservoir driving tumor initiation, therapeutic resistance, and relapse. These stem-like cells exhibit self-renewal capacity, multilineage differentiation potential, metabolic adaptability, and resistance to genotoxic stress, enabling them to survive cytotoxic therapy and regenerate tumor bulk. Notably, GSCs are not static entities but dynamic cellular states influenced by genetic alterations, epigenetic regulation, and microenvironmental cues. The central role of GSCs in GBM recurrence underscores why established approaches targeting rapidly proliferating tumor cells have yielded only transient advances. Following surgical resection, radiotherapy, and temozolomide treatment, residual GSCs residing within protective niches, particularly hypoxic and perivascular microenvironments, can re-enter the cell cycle and repopulate the tumor. Their boosted DNA repair capacity, expression of drug efflux transporters, immune evasion strategies, and metabolic flexibility collectively confer a survival advantage. Moreover, phenotypic plasticity allows segregated tumor cells to re-acquire stem-like properties under therapeutic pressure, further thwarting eradication attempts. Given this complexity, it is increasingly apparent that monotherapies targeting a single signaling pathway are unlikely to yield durable responses. The extensive crosstalk among developmental pathways (Notch, Wnt, Hedgehog), oncogenic drivers (RTKs, PI3K/AKT/mTOR, STAT3), and epigenetic regulators creates vigorous, compensatory networks that can sustain stemness even when one axis is inhibited. Therefore, combinatorial approaches targeting multiple intersections and concurrently disrupting supportive microenvironmental niches are cardinal.

Interventions that modulate the environment, including hypoxia signaling, vascular interactions, immune suppression, and extracellular matrix dynamics, may undermine the protective ecosystem that shields GSCs from therapy. Integration of metabolic targeting, immune modulation, and pathway inhibition may further sensitize GSCs to standard-of-care treatments. Advances in nanomedicine and blood–brain barrier-penetrating delivery systems provide additional opportunities to enhance drug bioavailability and selectively target stem-like populations within the central nervous system. Promisingly, emerging technologies—including single-cell and spatial transcriptomics, multi-omics integration, and patient-derived organoid models—are reshaping our understanding of GSC heterogeneity and therapeutic susceptibility. These tools enable the identification of discrete stem-like subpopulations and context-specific dependencies that can inform precision medicine strategies. Biomarker-driven clinical trial designs and adaptive therapeutic platforms will be critical to translating these insights into improved patient outcomes. Although substantial challenges remain, the paradigm shift toward targeting GSCs offers renewed hope for meaningful therapeutic progression. By combining molecularly informed interventions with strategies that disrupt tumor-supportive niches and overcome adaptive resistance, it may be possible to achieve more robust tumor control. Eventually, incorporating GSC-directed therapies into multimodal treatment regimens holds promise for enhancing survival and improving quality of life for GBM patients.

## Figures and Tables

**Table 1:** Glioblastoma stem cell (GSC) subtypes and key markers.

GSC Subtype	Key Markers	Functional Features	References
Classical/Proliferative	CD133^+^, SOX2^+^, OLIG2^+^	High proliferative capacity, prone to neurosphere formation, drives tumor growth	[24,30]
Mesenchymal/Invasive	CD44^+^, YKL-40^+^, Nestin^+^	Enhanced migration/invasion, therapy-resistant, associated with hypoxic/perivascular niches	[12,31]
Proneural/Neural Progenitor-like	SOX2^+^, OLIG2^+^, PDGFRA^+^	Stem-like plasticity, capable of differentiation into neural lineages, and moderate therapy resistance	[4,26]
Quiescent/Slow-Cycling	HES1^+^, BMI1^+^, CD133 low	Dormant cells, evade cytotoxic therapies, reservoir for tumor relapse	[10,26]
Hypoxia-Adapted	HIF1α^+^, CA9^+^, VEGF^+^	Survival under low oxygen, enhanced glycolytic metabolism, radioresistant	[15,32]
CD133-Negative Stem-Like	SOX2^+^, Nestin^+^, OLIG2^+^ (CD133^−^)	Maintains tumor-initiating capacity despite lack of CD133, shows phenotypic plasticity	[11,12]

**Table 2:** Core signaling networks governing glioblastoma stem cell (GSC) maintenance.

Pathway Category	Pathway/Key Components	Functional Role in GSCs	Therapeutic Insights/Inhibitors	References
Developmental Signaling	Notch (Notch1–4, Jagged, Delta, NICD, HES/HEY)	Self-renewal, asymmetric division, stemness maintenance, therapy resistance; integrates hypoxia cues via HIF-1α	γ-secretase inhibitors (GSIs), combination with radiotherapy/PI3K inhibitors; toxicity and compensatory pathways limit efficacy	[27,57,58]
	Wnt/β-catenin (Frizzled, LRP5/6, β-catenin, TCF/LEF, c-MYC, SOX2, NANOG)	Promotes proliferation, stemness, mesenchymal-like invasive phenotypes, and therapy resistance	Small-molecule inhibitors targeting porcupine, tankyrase, β-catenin–TCF; pathway redundancy is a challenge	[4,26,31]
	Hedgehog (Shh, PTCH, SMO, GLI1/2)	Maintains stemness, cell cycle progression, survival; crosstalk with Notch/Wnt	SMO inhibitors (vismodegib); direct GLI inhibitors in development; limited BBB penetration in GBM	[59,60]
Oncogenic/Survival Pathways	PI3K/AKT/mTOR	Self-renewal, proliferation, metabolic adaptation, resistance to TMZ/radiation	mTOR inhibitors (rapalogs), dual PI3K/mTOR inhibitors; feedback loops limit single-agent efficacy	[61,62,63]
	STAT3	Regulates SOX2, c-MYC, anti-apoptotic genes; promotes immune evasion (PD-L1)	STAT3 inhibitors (WP1066, napabucasin); combination with immunotherapies under investigation	[64,65]
	Receptor Tyrosine Kinases (EGFR, EGFRvIII, PDGFRA)	Drives proliferation, survival, stemness; heterogeneous expression enhances therapy resistance	RTK inhibitors (erlotinib, gefitinib, dasatinib); limited efficacy due to redundancy and tumor heterogeneity	[4,66,67]

**Table 3:** Molecular and functional mechanisms underlying GSC stemness and therapy resistance. The upward arrow (↑) represents a significant increase (or upregulation).

Mechanism	Key Features in GSCs	Functional Consequences	Representative References
DNA Damage Response (DDR)	↑ ATM/ATR signaling, ↑ Chk1/Chk2 activation, ↑ RAD51, BRCA1/2, γH2AX	Efficient repair of double-strand breaks, radioresistance, survival under genotoxic stress	[8,10,56]
Drug Efflux Transporters	↑ ABCG2, ↑ ABCB1/P-gp, high side-population activity	Active efflux of chemotherapeutics, TMZ resistance, reduced intracellular drug accumulation	[32,46]
Quiescence/Slow-Cycling State	Dormant cell subpopulations, regulated by Notch, TGF-β, BMP, HIF	Reduced susceptibility to cytotoxic therapies, reservoir for tumor recurrence	[10,17,26]
Metabolic Plasticity	Flexibly switch between glycolysis and OXPHOS; use fatty acid oxidation and glutamine metabolism; hypoxia-adapted glycolytic shift	Survival under nutrient and oxygen stress, therapy resistance	[19,50]
Temozolomide (TMZ) Resistance	MGMT expression/methylation, enhanced BER/MMR, anti-apoptotic signaling	Reduced TMZ efficacy, survival of GSCs post-chemotherapy	[8,10,54]
Radiation Resistance	Enhanced DDR, ROS scavenging, PI3K/AKT and STAT3 activation, hypoxia-mediated protection	Radioresistance, survival after ionizing radiation	[8,19,56]
Epigenetic Regulation	DNA methylation, histone acetylation/methylation, chromatin remodeling	Modulates stemness gene expression, transcriptional plasticity, therapy evasion	[16,54]
Non-coding RNAs	miRNAs (miR-21, miR-34a), lncRNAs (HOTAIR, MALAT1, NEAT1)	Regulation of stemness, proliferation, drug resistance, chromatin modification	[87,88,89]
Super-Enhancer Networks	Clusters of enhancers driving SOX2, OLIG2, POU3F2 expression; BRD4/Mediator occupancy	Sustains transcriptional programs for stemness, potential therapeutic vulnerability	[90,91]

**Table 4:** Emerging therapeutic strategies targeting glioblastoma stem cells (GSCs).

Strategy Category	Approach/Target	Mechanism/Action	Preclinical/Clinical Status	Challenges/ Limitations	Representative References
Targeting Developmental and Oncogenic Pathways	γ-Secretase Inhibitors (Notch)	Inhibit Notch intracellular domain cleavage → reduces stemness, neurosphere formation	Sensitizes GSCs to radiation in vitro	GI toxicity, compensatory pathway activation; combination therapies under investigation	[47,108]
	Wnt Inhibitors	Block β-catenin–TCF transcription, inhibit proliferation/self-renewal	Reduces GSC self-renewal	Pathway redundancy, context-dependent functions, systemic toxicity	[31,70,73]
	Hedgehog Inhibitors (SMO/GLI)	Suppress GLI-mediated transcription, impair stemness	Preclinical GSC reduction	Poor BBB penetration, GLI activation independent of SMO	[60,76,77]
	PI3K/mTOR Inhibitors	Inhibit survival/proliferation signaling	Reduces proliferation in vitro	Feedback loops, metabolic adaptation; combination therapies explored	[56,78,80]
	STAT3 Inhibitors	Block STAT3-mediated stemness, survival, immune evasion	Xenograft tumor suppression	Heterogeneous responses; best in combination with immunotherapy	[26,81,82]
Differentiation Therapy	BMPs (e.g., BMP4)	Promote astrocytic differentiation, suppress stemness	Preclinical reduction in tumorigenicity	Resistance via epigenetic silencing	[16,91,134]
	Retinoic Acid (ATRA)	Induces differentiation, reduces proliferation	Preclinical models	Poor CNS penetration, rapid metabolism	[135]
	Epigenetic Modulators (HDAC, EZH2, BET inhibitors)	Reprogram transcriptional state, promote differentiation	Sensitize tumors to radiation; reduce stemness	Off-target effects, systemic toxicity	[16,91]
Immunotherapeutic Strategies	CAR-T Cells	Target GSC antigens (EGFRvIII, IL13Rα2, HER2)	Early-phase clinical trials; transient tumor regression	Antigen heterogeneity, immune suppression	[136]
	Dendritic Cell Vaccines	Adaptive immune activation	Clinical trials; survival benefit in subsets	Need larger trials; variable immune response	[137]
	Immune Checkpoint Blockade (PD-1/PD-L1)	Release T-cell inhibition	Clinical trials in GBM; limited success	Immunosuppressive microenvironment, low TMB	[138]
	Oncolytic Viruses	Selective infection and lysis of GSCs, stimulate immunity	Preclinical and early-phase trials	Delivery, antiviral immunity, BBB limitations	[139]
Metabolic Targeting	OXPHOS Inhibitors (e.g., metformin, phenformin)	Impair mitochondrial respiration	Reduce stemness in OXPHOS-dependent GSCs	Off-target systemic effects, heterogeneity in metabolic reliance	[140,141]
	Glycolysis Inhibitors (e.g., HK2 inhibitors)	Block glucose metabolism in hypoxia-adapted cells	Reduced survival	Systemic toxicity, pathway redundancy	[50]
	Redox Modulators	Inhibit glutathione/NADPH, ROS accumulation	Sensitize GSCs to TMZ/radiation	Risk to normal cells	[142,143]
Nanomedicine-Based Drug Delivery	BBB-Penetrating Nanoparticles	Ligand-mediated transcytosis (transferrin, angiopep-2)	Enhance CNS drug delivery	Heterogeneous BBB permeability	[144]
	Liposomal/Polymeric Systems	Controlled drug release; co-delivery of chemotherapeutics and inhibitors	Improve pharmacokinetics	Limited targeting specificity	[145]
	CD133-Targeted Nanoparticles	Antibody-conjugated delivery to stem-like cells	Enrich drug delivery to GSCs	Marker heterogeneity limits efficacy	[146]
	Combination Nanotherapeutics	Multi-functional delivery (siRNA, small molecules, immunomodulators)	Target signaling, metabolism, and immune evasion	Complexity of formulation, translational barriers	[147]

## Data Availability

No new data were created or analyzed in this study. Data sharing is not applicable to this article.

## Abbreviations

The following abbreviations are used in this manuscript:

ABC	ATP-binding cassette (transporter)
ATRA	All-trans retinoic acid
BBB	Blood–brain barrier
BET	Bromodomain and extra-terminal domain
BMP	Bone morphogenetic protein
CAR	Chimeric antigen receptor
CNS	Central nervous system
DDR	DNA damage response
DC	Dendritic cell
EMT	Epithelial–mesenchymal transition
EGFRvIII	Epidermal growth factor receptor variant III
GSC	Glioblastoma stem cell
GBM	Glioblastoma
GLI	GLI family zinc finger transcription factor
HDAC	Histone deacetylase
HH	Hedgehog (signaling pathway)
IL-6R	Interleukin-6 receptor
IL13Rα2	Interleukin-13 receptor alpha 2
mTOR	Mechanistic target of rapamycin
ncRNA	Non-coding RNA
NK	Natural killer (cell)
OXPHOS	Oxidative phosphorylation
PD-1	Programmed cell death protein 1
PD-L1	Programmed death-ligand 1
PI3K	Phosphoinositide 3-kinase
PTCH	Patched receptor
RAPALOG	Rapamycin analog
RNA	Ribonucleic acid
RTK	Receptor tyrosine kinase
SMO	Smoothened receptor
STAT3	Signal transducer and activator of transcription 3
SHH	Sonic Hedgehog
SOX2	SRY-box transcription factor 2
TCF/LEF	T-cell factor/Lymphoid enhancer-binding factor
TMZ	Temozolomide
VEGF	Vascular endothelial growth factor
VEGFR	Vascular endothelial growth factor receptor
ZEB1	Zinc finger E-box-binding homeobox 1
SNAIL	Zinc finger protein SNAI1
TWIST	TWIST-related protein 1
